# Effects of fecal microbiota transplantation in metabolic syndrome: A meta-analysis of randomized controlled trials

**DOI:** 10.1371/journal.pone.0288718

**Published:** 2023-07-20

**Authors:** Bo Qiu, JiaXu Liang, Cong Li

**Affiliations:** 1 International Doctoral School, University of Seville Faculty of Medicine, Seville, Spain; 2 Department of Diagnostic Radiology, The Fifth Clinical Medical College of Henan University of Chinese Medicine (Zhengzhou People’s Hospital), Zhengzhou, China; 3 Department of Endocrinology of North District, The Second Affiliated Hospital of Zhengzhou University, Zhengzhou, China; Jinnah Sindh Medical University, PAKISTAN

## Abstract

**Objective:**

The prevalence of obesity and type 2 diabetes is rapidly increasing worldwide, posing serious threats to human health. This study aimed to evaluate the role of FMT in the treatment of obesity and/or metabolic syndrome and its impact on clinically important parameters.

**Methods:**

We searched Medline, Embase, and Cochrane Library databases up to April 31, 2022 and further assessed articles that met the eligibility criteria. Mean differences and 95% confidence intervals were used to analyze continuous data. The I^2^ statistic was used to measure study heterogeneity. Univariate meta-regression or subgroup analyses were performed to explore the covariates that might contribute to heterogeneity. Potential publication bias was assessed using the Egger’s test. We used the GRADEpro guideline development tool to assess the quality of the evidence.

**Results:**

Nine studies, comprising 303 participants, were included in the meta-analysis. In the short-term outcomes (<6 weeks after FMT), compared with the placebo group, patients in the FMT group had lower FBG (MD = -0.12 mmol/L, 95% Cl: -0.23, -0.01), HbA1c (MD = -0.37 mmol/mol, 95%Cl: -0.73, -0.01), and insulin levels (MD = -24.77 mmol/L, 95% Cl: -37.60, -11.94), and higher HDL cholesterol levels (MD = 0.07 mmol/L, 95% Cl: 0.02, 0.11).

**Conclusions:**

FMT, as an adjunctive therapy, does not produce any serious adverse effects and may be useful in the treatment of metabolic syndrome, especially in improving HbA1c, insulin sensitivity, and HDL cholesterol. However, there was no significant difference between the FMT group and the placebo group in terms of weight reduction.

## Introduction

Since the 1970s, obesity has escalated into a global epidemic, with obesity rates tripling worldwide, and affecting approximately one-tenth of the global adult population. With the increased prevalence and severity of the disease, obesity has become a major underlying risk factor for chronic diseases, such as type 2 diabetes mellitus (T2DM), cardiovascular disease, metabolic syndrome (MetS), non-alcoholic fatty liver disease (NAFLD), and cancer, increasing morbidity and mortality [1]. According to statistics released by the International Diabetes Federation (IDF), the number of people with diabetes will increase to 592 million by 2035, the number of adults with diabetes will increase by 55%, and over 80% of people with type 2 diabetes will be obese [2]. Most conventional treatments for obesity and obesity-related diseases are unsuccessful. Therefore, there is an urgent need to develop novel treatment strategies.

In recent years, fecal microbiome transfer (FMT) has been established as an effective treatment for recurrent Clostridioides difficile infection (CDI) because it can re-establish the intestinal microecosystem [3]. FMT has also been shown to be beneficial in the treatment of many other diseases such as irritable bowel syndrome (IBS), inflammatory bowel disease (IBD), and other gastrointestinal disorders [4–10]. In addition to genetic factors and lifestyle, the composition of the gut microbiome plays an important role in obesity and insulin resistance [11–13]. In the last decade, several studies have reported that alterations in gut microbiome composition are associated with obesity, glucose metabolism, and insulin sensitivity [14, 15]. In obese and diabetic patients, insulin sensitivity can be improved by establishing normal gut microbiome ratios, altering low-grade chronic inflammatory responses, correcting disturbances in bile acid metabolism, and interfering with short-chain fatty acid (SCFA) production to modulate the gut microecosystem [16–18]. Animal studies have successfully altered body phenotypes by using FMT. Pioneering experiments in mice have shown that obese and lean phenotypes can be transferred through the fecal microbiome of human donors [19–21]. This evidence highlights the possibility of using FMT as a therapeutic modality for human obesity.

FMT can reverse the pathological microecosystem in the intestinal tract and use the gut microbiome as a new target to treat metabolic diseases, such as diabetes, which will become a unique therapeutic idea. However, the sample sizes in some studies were too small, the statistical power was too low to predict the outcome of a comprehensive study, and there was a lack of research on the long-term effects of FMT. However, the use of FMT to alter the microbiome and improve clinically important parameters remains controversial. We conducted a systematic review and meta-analysis of randomized clinical trials (RCTs). To assess the role of FMT in the treatment of obesity with or without metabolic syndrome and its impact on clinically important parameters.

## Materials and methods

The Preferred Reporting Items for Systematic Reviews and Meta-Analyses (PRISMA) guidelines were adhered to as a methodological template for this review [22] (S1 Table).

### Literature search strategy

Two investigators (B. Q and JX. L) independently searched MEDLINE (using PUBMED as the search engine), EMBASE, and Cochrane Library. Databases were used to identify suitable studies published until May 31, 2022. MeSH terms and keywords were used, and the search terms included: “fecal microbiota transplantation,” “fecal microbial transplant,” “fecal microbiota transfer,” “FMT,” “obesity,” “diabetes,” and “metabolic syndrome”. The search was limited to publications on human subjects in English. A manual search was conducted using references listed in the original articles and the review articles retrieved. Two investigators collected results separately.

### Inclusion criteria

1. Randomized clinical trials (RCT);
2. Diagnosis of obesity and/or metabolic syndrome. (Obesity: defined as BMI ≥ 30 kg/m^2^)
3. The following related data were extracted: weight, body mass index (BMI), fasting blood glucose (FBG), hemoglobin A1C (HbA1c), HOMA-IR (Homeostatic Model Assessment for Insulin Resistance), insulin, cholesterol (total/LDL/HDL) and triglyceride,

### Exclusion criteria

1. Duplicate reports
2. Studies conducted on animals
3. Systematic reviews or meta-analyses
4. Case-control and cohort study

### Data extraction

For each included study, all data elements uniformly reported across most studies were extracted by two reviewers (B. Q and JX. L) and cross-verified by a third (C. L). When the same population was published in several journals, only the most informative articles or complete studies were retained to avoid duplication. The following information was extracted from each study: first author, publication year, patient characteristics, number of patients, method of FMT/placebo use, preoperative preparation, follow-up, and study results.

### Definition of short-term /long-term outcomes

We considered short-term outcomes to be those that occurred within six weeks of intervention. Long-term outcomes were defined as those that occurred ≥ 12 weeks after the intervention. To analyze the effects of the intervention, we divided them into two groups: short-term and long-term effects, and analyzed the means of differences in clinically meaningful parameters separately.

### Risk of bias assessment

The Cochrane risk-of-bias tool was used to assess the risk of bias in the randomized trials [23]. The quality of the evidence was assessed using the GRADEpro guideline development tool. Five items were assessed to obtain the quality of evidence: risk of bias, inconsistency, indirectness, imprecision, and publication bias. The quality of evidence can be classified as very low, low, moderate or high. Each included article was independently assessed by two authors using this tool, and disagreements between the two authors were resolved by consensus.

### Statistical analysis

Mean differences (MD) and 95% confidence intervals (CIs) were used to analyze continuous data. The methods described by Luo et al. [24] and Wan et al. [25] were used to estimate the mean and standard deviation, with medians and ranges, to make the data suitable for meta-analysis. For the selection of effect sizes, the choice of weight mean differences (WMD) is appropriate if different studies use the same units of measurement for the observed continuous-type metrics, or standardized mean differences (SMD) if the units of measurement differ and/or the mean varies significantly [26].

The I^2^ statistic was used to measure the study heterogeneity, with I^2^ ≥ 50% indicating significant heterogeneity. A fixed-effects model was used when heterogeneity was not significant; otherwise, a random-effects model was applied. Univariate meta-regression or subgroup analyses were performed to explore covariates that might contribute to heterogeneity based on the following predetermined characteristics: 1. years of publication (earlier than 2020 vs later than 2020); 2. race (European vs. non-European), and 3. method of FMT intervention (oral vs. non-oral). Sensitivity analysis was performed to determine whether there was an undue influence of a single study on the combined study results [27].

We assessed potential publication bias using Begg’s test and Egger’s test, with P >0.05 indicating no publication bias. All statistical analyses were performed using Stata version 15 (Stata Corp, College Station, Texas, USA) and RevMan 5.4 (The Cochrane Collaboration, Oxford, UK).

## Results

### Characteristics of the included studies

**Fig 1:**
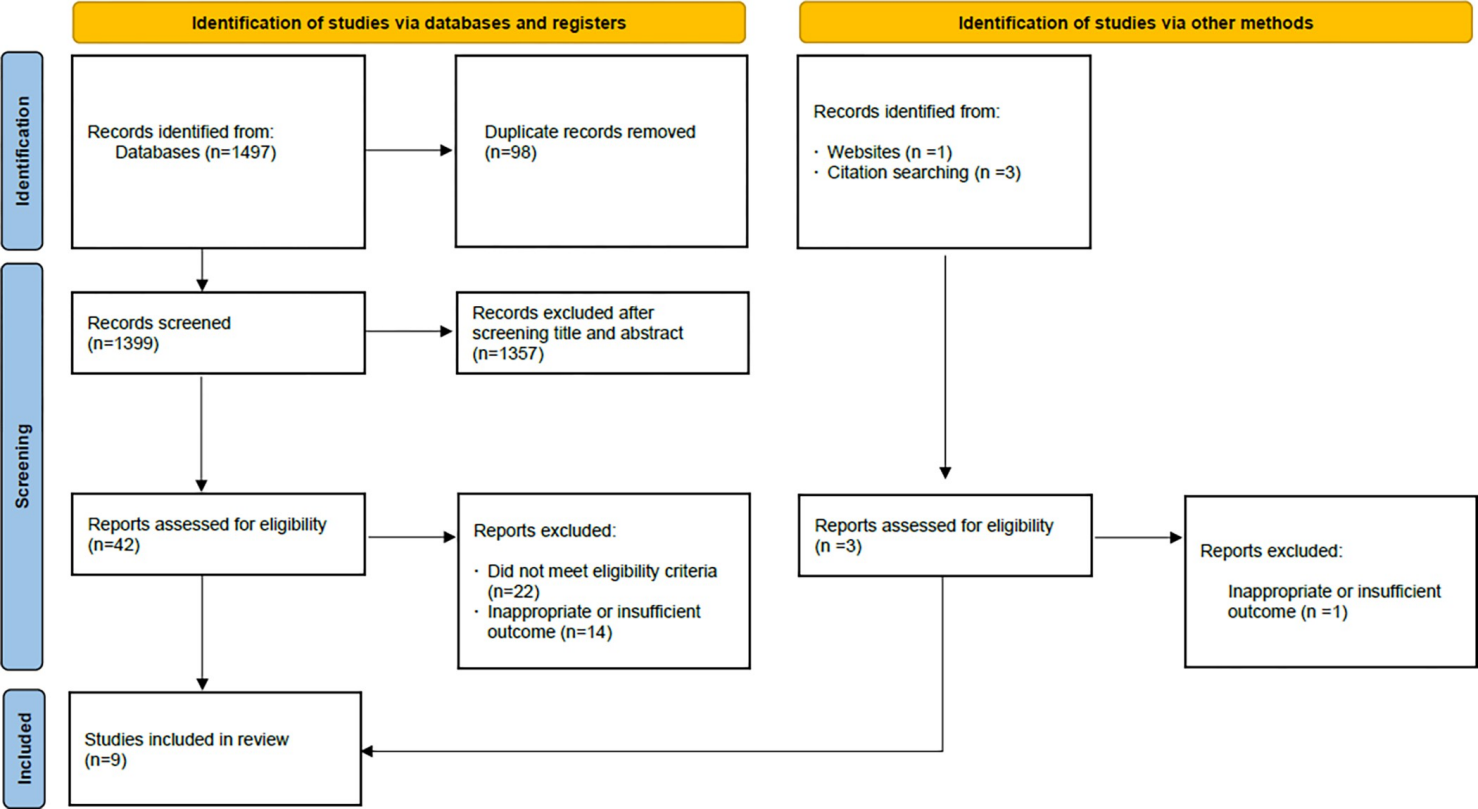
Study identification and selection flowchart.

**Fig 2:**
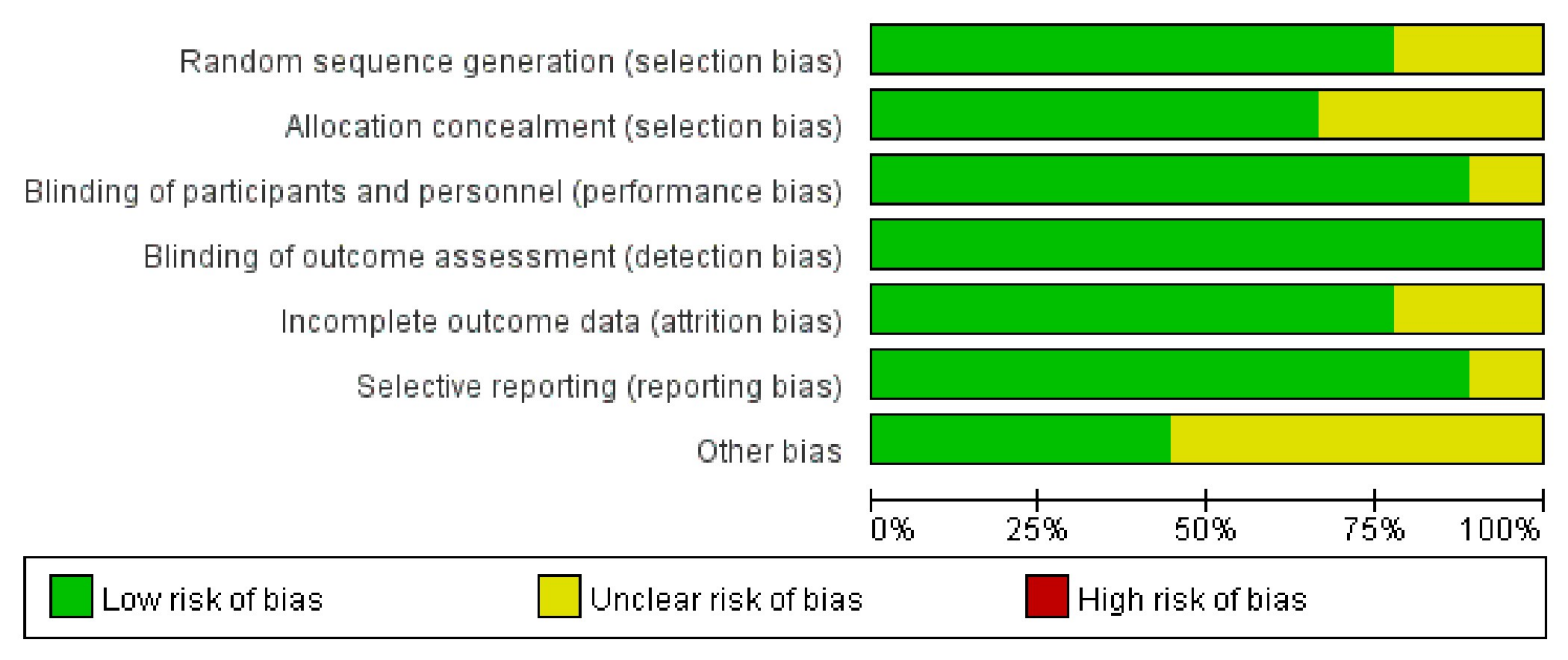
Evaluation of study quality.

Of the 1428 articles identified through the systematic search, nine studies [28–36] with 303 participants were finally included in our meta-analysis. Fig 1 showed the identification and selection of studies. All the included studies were RCTs. Among these nine studies, two [28, 30] evaluated the effect of FMT in obese patients (BMI ≥ 30 kg/m^2^) without metabolic syndrome, one [33] only evaluated the effect of FMT in patients with type 2 diabetes without BMI, five studies [29, 31, 32, 34, 35] assessed the role of FMT in patients with metabolic syndrome and obesity, and one study [36] included patients with normal BMI and type I diabetes. Four studies [29, 32, 34, 36] performed FMT using a nasoduodenal tube and five studies [28, 30, 31, 33, 35] used oral capsules. Two of these studies [31, 33] combined FMT with oral capsules in a specific diet. All studies used stool from healthy lean donors for FMT; however, in one study [32] donors were vegetarians. The main characteristics of the included studies are summarized in Table 1. Before data analysis and synthesis, the Cochrane risk of bias tool was used to assess the quality of the studies, as shown in Fig 2. Five studies were at risk of other bias because they did not report the history of cholecystectomy, as animal studies have shown that the composition of the gut microbiota is influenced by a variety of factors, including bile acid composition [28, 31, 33, 35, 36].

**Table 1:** Baseline characteristics of the included studies.

Author (year)	Country	Participants	Case (n)	Age (years)	BMI (kg/m^2^)	Intervention	Follow up
Allegretti (2019) ^[^^28^^]^	United Kingdom	Female participants, BMI ≥ 35 kg/m^2^ without metabolic syndrome	22: 11 FMT 11 Placebo	FMT: 44.5±14.4 Placebo: 43.2±13.8	FMT: 41.1±5.0 Placebo: 40.4±4.7	FMT/ Placebo capsules	12 weeks
Kootte 2017 ^[^^29^^]^	Netherlands	Male patients aged 21–69 years with metabolic syndrome	38: 26 Allogenic FMT 12 Autologous FMT	Autologous: 54 (49–58) Allogenic: 54 (49–60)	Autologous: 35.8 (33.1–40.4) Allogenic: 33.8 (32.5–35.7)	FMT/ autologous FMT was infused through the nasoduodenal tube.	18 weeks
Leong 2020 ^[^^30^^]^	New Zealand	Patients aged 14–18 years, BMI ≥ 30 kg/m^2^ and without chronic diseases.	87: 42 FMT 45 Placebo	FMT: 17.3±1.5 Placebo: 17.1±1.4	FMT: 38.6±5.9 Placebo: 36.9±4.6	FMT/ Placebo capsules	26 weeks
Mocanu 2021 ^[^^31^^]^	Canada	Patients aged 18–65 years, BMI ≥ 30 kg/m^2^ with metabolic syndrome	61: 29 FMT 32 Placebo	FMT: 47.3±11.0 Placebo: 48.4±9.6	FMT: 46.3±6.6 Placebo: 44.5±7.2	FMT/ Placebo capsules coupled with fiber supplementation	12 weeks
Smits (2018) ^[^^32^^]^	Netherlands	Male patients aged 21–69 years, BMI ≥ 30 kg/m^2^ with metabolic syndrome	20: 10 Allogenic FMT 10 Autologous FMT	Autologous: 57.7±8.5 Allogenic: 52.3±7.4	Autologous: 33.8±4.0 Allogenic: 33.9±3.9	FMT/ autologous FMT was infused through the nasoduodenal tube.	2 weeks
Su 2022 ^[^^33^^]^	China	Patients aged 41–76 years, with type 2 diabetes	13: 5 FMT 8 Placebo	FMT: 57±13.2 Placebo: 60.4±12.0	FMT: 25.2±5.0 Placebo: 24.8±3.0	received the PPW^b^ formulation only, or coupled with FMT capsules	90 days
Vrieze 2012 ^[^^34^^]^	Netherlands	Male patients, BMI ≥ 30 kg/m^2^ with metabolic syndrome	18: 9 Allogenic FMT 9 Autologous FMT	Autologous: 53±3 Allogenic: 47±4	Autologous: 35.6±1.5 Allogenic: 35.7±1.5	FMT/ autologous FMT was infused through the gastroduodenal tube	6 weeks
Yu 2020 ^[^^35^^]^	USA	Patients aged 25–60 years, BMI ≥ 30 kg/m^2^ and mild to moderate insulin resistance^c^	24: 12 FMT 12 Placebo	FMT: 42.5±8.4 Placebo: 38.5±8.8	FMT: 38.8±6.7 Placebo: 41.3±5.1	FMT/ Placebo capsules	12 weeks
Groot 2020 ^[^^36^^]^	Netherlands	Patients aged 18–35 years with normal BMI, type 1 diabetes	20: 10 Allogenic FM 10 Autologous FMT	Autologous: 25.0±3.5 Allogenic: 24.3±5.4	Autologous: 23.0±2.0 Allogenic: 21.8±2.5	FMT/ autologous FMT was infused through the nasoduodenal tube.	12 months

Data are depicted as mean±SD or median (interquartile range), depending on their distribution. BMI, body mass index (calculated as weight in kilograms divided by height in meters squared); FMT, fecal microbiota transplantation.

a. FMT-LF group: FMT and low-fermentable fiber.

b. PPW: diet consisting of probiotics, prebiotics and whole grains.

c. mild to moderate insulin resistance: homeostatic model assessment of insulin resistance (HOMA-IR) between 2.0 and 8.0.

### Short-term outcomes

**Fig 3:**
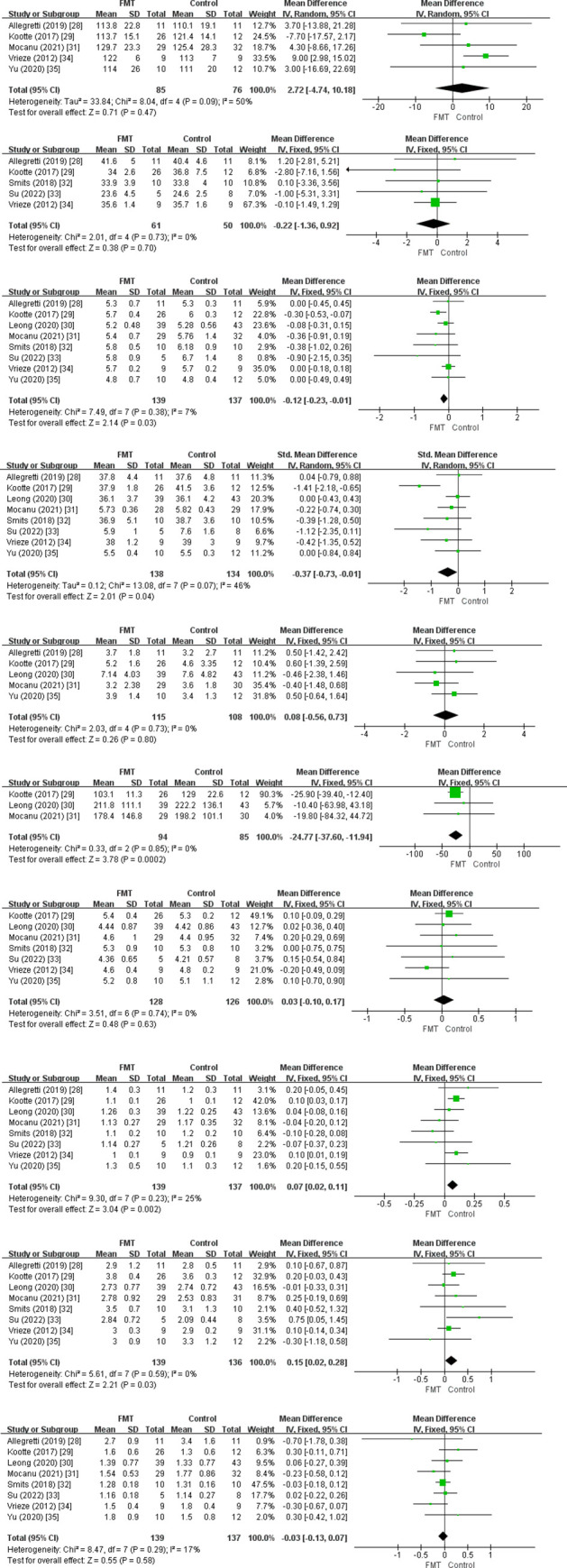
Forest plot of short-term factor results. 1) Weight (Kg), 2) BMI (Kg/m^2^), 3) Fasting glucose (mmol/L), 4) HbA1C, 5) HOMA-IR, 6) Insulin (pmol/L), 7) Cholesterol (mmol/L), 8) HDL (mmol/L), 9) LDL (mmol/L), 10) Triglycerides (mmol/L).

A total of 303 patients were included and analyzed to determine the significance of FMT in terms of metabolic syndrome-related efficacy outcomes. Statistically significant FBG (MD = -0.12 mmol/L, 95%Cl: -0.23, -0.01, SD: ±0.04, I^2^ = 7%), HbA1c (MD = -0.37 mmol/mol, 95%Cl: -0.73, -0.01, SD: ±0.13, I^2^ = 46%), HDL cholesterol (MD = 0.07 mmol/L, 95%Cl: 0.02, 0.11, SD: ±0.02, I^2^ = 25%), and insulin levels (MD = -24.77 pmol/L, 95%Cl: -37.60, -11.94, SD: ±4.76, I^2^ = 0%) were achieved with FMT in the short term. Compared with the placebo group, patients in the FMT group had lower FBG, HbA1c, and insulin levels, and higher HDL cholesterol levels. Weight (MD = 2.72 Kg, 95%Cl: -4.74, 10.18, SD: ±11.16, I^2^ = 50%), BMI (MD = -0.22 Kg/m^2^, 95%Cl: -1.36, 0.92, SD: ±1.71, I^2^ = 0%), HOMA-IR (MD = 0.08, 95%Cl: -0.56, 0.73, SD: ±0.96, I^2^ = 0%), total cholesterol (MD = 0.03 mmol/L, 95%Cl: -0.10, 0.17, SD: ±0.05, I^2^ = 0%), LDL cholesterol (MD = 0.13 mmol/L, 95%Cl: -0.03, 0.28, SD: ±0.23, I^2^ = 0%), and triglycerides (MD = -0.03 mmol/L, 95%Cl: -0.13, 0.07, SD: ±0.15, I^2^ = 17%) did not different between the two groups (Fig 3).

### Analysis of subgroups based on the method of FMT use

We performed a subgroup analysis of the two different ways of FMT use (Capsules VS. Nasoduodenal tube). There was no significant difference between the FMT group and the placebo group in each parameter by way of oral capsule administration. In contrast, the mean HbA1C and insulin levels were slightly lower and the mean HDL levels were slightly higher in the FMT group by the nasoduodenal tube injection (Table 2).

**Table 2:** Subgroup analysis based on FMT use method in short-term outcomes.

Category	Subgroup	MD	95%Cl	I^2^
Weight	Capsules	3.85	-5.37, 13.07	0%
	Nasoduodenal tube	1.13	-15.21, 17.46	50%
BMI	Capsules	0.18	-2.76, 3.11	0%
	Nasoduodenal tube	-0.29	-1.53, 0.94	0%
Fasting glucose	Capsules	-0.10	-0.28, 0.07	0%
	Nasoduodenal tube	-0.17	-0.42, 0.08	57%
HbA1C	Capsules	-0.11	-0.39, 0.17	0%
	Nasoduodenal tube	-0.83	-1.32, -0.34	50%
HOMA-IR	Capsules	0.02	-0.65, 0.70	0%
	Nasoduodenal tube	0.6	-1.39, 2.59	------
Insulin	Capsules	-14.24	-55.46, 26.98	0%
	Nasoduodenal tube	-25.90	-39.40, -12.40	------
Cholesterol	Capsules	0.10	-0.16, 0.36	0%
	Nasoduodenal tube	0.01	-0.15, 0.17	30%
LDLc	Capsules	0.13	-0.10, 0.35	19%
	Nasoduodenal tube	0.13	-0.09, 0.34	0%
HDLc	Capsules	0.04	-0.05, 0.12	0%
	Nasoduodenal tube	0.08	0.03, 0.13	56%
Triglycerides	Capsules	-0.03	-0.19, 0.14	0%
	Nasoduodenal tube	-0.03	-0.16, 0.10	56%

BMI: Body mass index; HbA1c: Hemoglobin A1c (glycated hemoglobin); HOMA-IR: Homeostatic model assessment of insulin resistance; LDL: Low density lipoprotein; HDL: High density lipoprotein

### Long-term outcomes

**Fig 4:**
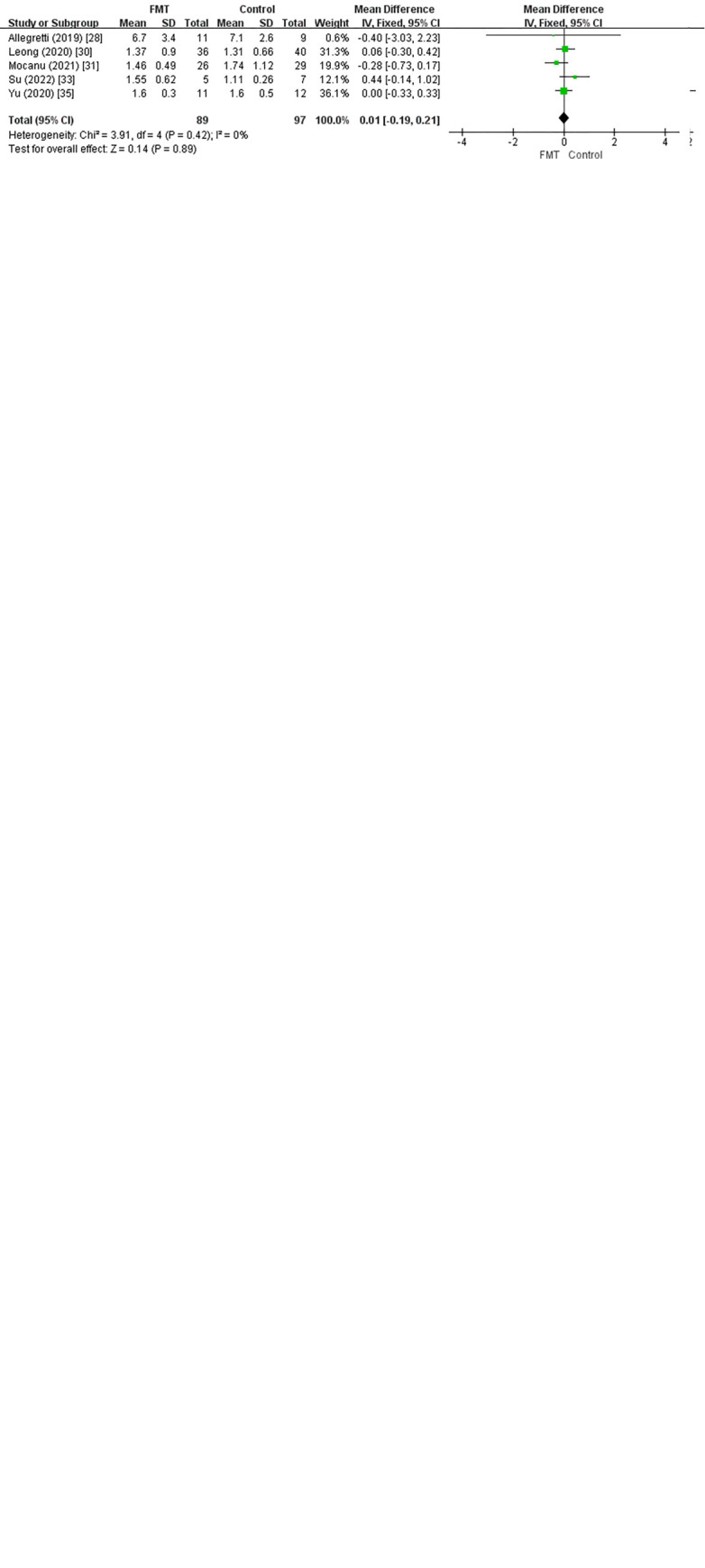
Forest plot of long-term factor result. 1) Weight (Kg), 2) BMI (Kg/m^2^), 3) Fasting glucose (mmol/L), 4) HbA1C, 5) HOMA-IR, 6) Insulin (pmol/L), 7) Cholesterol (mmol/L), 8) HDL (mmol/L), 9) LDL (mmol/L), 10) Triglycerides (mmol/L).

We found no difference between the FMT and control groups through analysis of the mean differences in clinically significant parameters (Fig 4), except for a slight decrease in HbA1c at 12 weeks in the FMT group compared to the placebo group in the study by Yu et al. [35].

### Adverse events

Only minor adverse events (AEs) were reported in the treatment group. Two studies [28, 35] reported fever, headache, nausea/vomiting, diarrhea, bloating, and abdominal pain. No significant differences in AEs were observed and no serious AEs were associated with FMT.

### Heterogeneity analysis

Significant heterogeneity was observed in weight (I^2^ = 50%) in the short-term outcome analyses. Hence, we conducted meta-regression and subgroup analyses to examine the sources of potential heterogeneity, but “Year of publication”, “Race”, and “FMT intervention methods” were not factors for heterogeneity (the p-values were 0.86,0.87 and 0.78, respectively). We then performed a sensitivity analysis by removing one study and recalculating the pooled estimates for the remaining studies, which showed that the pooled results were significantly affected by the Vrieze et al. [34]. Heterogeneity was 0% after its exclusion, but the result was still not significantly different between the two groups (MD = -1.53 Kg, 95%Cl: -8.27, 5.20).

### Publication bias

We performed a funnel plot to test for publication bias. Visual inspection of the funnel plot revealed asymmetry, which raises the possibility of publication bias (S1 Fig). Therefore, we used the Begg’s test and Egger’s to detect the risk of bias. All p-values of Begg’s test and Egger’s statistical test were greater than 0.05, and the results indicate that there was no publication bias among the studies included in the meta-analysis (Table 3).

**Table 3:** Results of Begg’s test and Egger’s statistical test.

	Short-term outcomes		Long-term outcomes	
Category	Begg’s Test	Egger’s test	Begg’s Test	Egger’s test
Weight	0.81	0.39	1.0	––
BMI	0.81	0.49	1.0	––
Fasting glucose	0.27	0.65	0.30	0.19
HbA1C	0.11	0.23	0.46	0.57
HOMA-IR	0.46	0.76	1.0	––
Insulin	1.0	––	1.0	––
Cholesterol	0.76	0.59	0.73	0.48
LDLc	0.17	0.18	0.46	0.54
HDLc	0.99	0.52	0.81	0.69
Triglycerides	0.71	0.83	0.81	0.38

BMI: Body mass index; HbA1c: Hemoglobin A1c (glycated hemoglobin); HOMA-IR: Homeostatic model assessment of insulin resistance; LDL: Low density lipoprotein; HDL: High density lipoprotein

### Quality of the evidence for the results

We used the GRADEpro guideline development tool to assess the quality of the evidence (Table 4 and S3 Table).

**Table 4:** Quality of evidence by Grading of Recommendations Assessment, Development and Evaluation (GRADE).

	Outcome	Studies	Participants	Quality of the evidence	GRADE	Importance
Short-term effects	Weight	5	161	⊕⊝⊝⊝	very low	IMPORTANT
	BMI	5	111	⊕⊕⊝⊝	low	IMPORTANT
	Fasting glucose	8	276	⊕⊕⊕⊝	moderate	IMPORTANT
	HbA1C	8	272	⊕⊕⊕⊝	moderate	IMPORTANT
	HOMA-IR	5	223	⊕⊕⊝⊝	low	IMPORTANT
	Insulin	3	179	⊕⊕⊕⊝	moderate	IMPORTANT
	Cholesterol	7	254	⊕⊕⊝⊝	low	IMPORTANT
	LDLc	8	278	⊕⊕⊝⊝	low	IMPORTANT
	HDLc	8	278	⊕⊕⊕⊝	moderate	IMPORTANT
	Triglycerides	8	276	⊕⊕⊝⊝	low	IMPORTANT
Long-term effects	Weight	2	43	⊕⊕⊝⊝	low	IMPORTANT
	BMI	2	90	⊕⊕⊝⊝	low	IMPORTANT
	Fasting glucose	3	111	⊕⊕⊝⊝	low	IMPORTANT
	HbA1C	5	149	⊕⊕⊝⊝	low	IMPORTANT
	HOMA-IR	2	77	⊕⊕⊝⊝	low	IMPORTANT
	Insulin	2	96	⊕⊕⊝⊝	low	IMPORTANT
	Cholesterol	4	166	⊕⊕⊝⊝	low	IMPORTANT
	LDLc	5	186	⊕⊕⊝⊝	low	IMPORTANT
	HDLc	5	186	⊕⊕⊝⊝	low	IMPORTANT
	Triglycerides	5	186	⊕⊕⊝⊝	low	IMPORTANT

BMI: Body mass index; HbA1c: Hemoglobin A1c (glycated hemoglobin); HOMA-IR: Homeostatic model assessment of insulin resistance; LDL: Low density lipoprotein; HDL: High density lipoprotein

## Discussion

Obesity and metabolic syndrome are global health epidemics of the 21st century, and current medical strategies have limited efficacy [37]. Several studies have reported that patients with obesity and metabolic syndrome have abnormal gut microbiota; therefore, the treatment of obesity and diabetes can be initiated by modulating the gut microbiota [38–40]. High-fat and high-sugar diets can lead to a large proliferation of Firmicutes and a decrease in Bacteroidetes; an altered ratio of Firmicutes to Bacteroidetes is associated with metabolic diseases such as obesity [41, 42]. Compared to lean individuals, the gut microbiota of obese individuals is more conducive to the production of energy-related molecules, particularly short-chain fatty acids of resistant starch origin. These molecules can generate additional energy through the citric acid cycle or participate in gluconeogenesis, lipid metabolism, and protein metabolism [43]. FMT transplants the functional gut microbiota from the feces of healthy individuals into the gastrointestinal tract of obese individuals to re-establish functional gut microbiota.

This meta-analysis investigated studies using FMT for the treatment of obesity and metabolic syndrome and basically concluded that the treatment was effective in the short term. At 2 to 6 weeks after the intervention, mean HbA1c and mean fasting glucose were lower in the FMT group than in the placebo group, although this was a small mean difference. However, mean insulin levels were significantly lower in the FMT group, suggesting a significant improvement in insulin sensitivity. One study [31] reported a significant improvement in HOMA2-IR after six weeks of FMT application. There are also studies [29, 34] that reported improved peripheral insulin sensitivity in the FMT group. Moreover, two studies even showed a small decrease in HbA1c after FMT intervention [29, 35]. FMT has been reported to treat obesity in mice [44], and Zhang et al. [45] showed that FMT improved some laboratory parameters (e.g., insulin sensitivity, glycated hemoglobin, etc.) in patients with metabolic syndrome, although none of the weight loss effects were significant. Therefore, these findings could prove that FMT is effective for glycemic control and improves insulin sensitivity, although the improvement is small.

There was epidemiological evidence of an association between systemic and/or local low-grade chronic inflammation and insulin resistance (IR) states. The development of IR is mainly associated with various pro-inflammatory cytokines, such as tumor necrosis factor-α (TNF-α), interleukin-6 (IL-6), and interleukin-1β (IL-1β) [46–48]. Additionally, the inflammatory marker C-reactive protein (CRP) is generally elevated in human IR states [49]. Several factors may contribute to the initiation and maintenance of tissue inflammation, such as diet, tissue microenvironment, and gut microbiota [50]. Chronic exposure to pro-inflammatory mediators causes cell-autonomous IR in liver, muscle and adipocytes [48]. Then whether the regulation of gut microbiota can improve the inflammatory response and IR has also become the outcome of interest in current studies. It has been demonstrated in several studies that fasting blood glucose levels were significantly reduced and IR and low inflammatory response were improved after treatment of diabetic patients with gut microbiota improvement. [51, 52].

There was also a small but statistically significant difference in HDL cholesterol levels between the two groups. After the short-term intervention, mean HDL cholesterol levels were higher in the FMT group than in the placebo group. A previous meta-analysis also reported a slightly higher mean HDL in the FMT group compared to the placebo group [53]. Although the quality of the evidence for this finding is low, the evidence accumulated from intervention studies using FMT suggests a possible association between FMT and changes in cholesterol metabolism. Intervention of high-fat diet-induced NAFLD in mice by transplanting fecal bacteria from normal mice in several animal model experiments resulted in lower body weight, lower blood lipids, improved liver function and reduced hepatic steatosis in NAFLD rats [54, 55]. However, there is still a lack of clinical trial results with a strong enough evidence level for evidence-based medicine.

The previously described benefits were not observed in the long-term group, and there were no significant differences between the FMT and placebo groups with respect to important obesity parameters (e.g., weight, BMI) in the short and long term. The small number of patients enrolled in the trial may explain why we did not observe any between-group differences in these parameters. In a study by Kootte et al. [29], long-term clinical effects, such as sensitivity to insulin and plasma metabolites were lacking at 18 weeks after allogeneic FMT. One hypothesis that could explain the return of gut microbiota composition to baseline conditions and the varying degrees of short- and long-term metabolic responses is that the host immune system develops resilience coupled with adherence to its own lifestyle, including diet and exercise [56]. In a study of mice with a tightly controlled diet, weight loss was associated with FMT treatment, and a poor diet may counteract the beneficial effects of FMT [19]. Overall, the findings of these studies and our results suggested that FMT may be effective in alleviating the features of metabolic syndrome. Extensive research is needed to reveal the specific pathophysiological roles of the gut microbiota in obesity and diabetes and to observe the mechanism and clinical efficacy of FMT in the treatment of metabolic-related diseases.

In several studies, the administration of FMT is preferred by using a duodenal infusion [29, 32, 34, 36]. The solution was infused within 6 hours of collection of fecal material through the nasoduodenal tube. In contrast to this method of transplantation, in order to prevent adverse events associated with physical delivery of FMT using nasojejunal tubes or colonoscopy, some trial groups have adopted a non-invasive method of FMT delivered by double wrapping using an encapsulated fecal microbiome to transport the contents to the intestine using a delayed-release hydroxypropyl methylcellulose capsule that remains intact as it passes through the stomach [57]. Notably, in our subgroup analysis, the improvement in HbA1C and HDL was more significant with the method of FMT infusion through the nasal-intestinal tube compared to the oral capsule method This may be due to the fact that fresh fecal suspension can be placed more precisely at the appropriate site by endoscopic injection. Combined with the fact that absorption of sugars and fatty acids is associated with obesity and insulin resistance, and that these substances are mainly absorbed in the small intestine, the best route to achieve FMT is via nasoduodenal tube infusion.

Significant heterogeneities of pooled specificity were still found among studies when we used the random-effects model in the pooling of data. This method might reduce the effect of heterogeneity but not abolish it. To explore the sources of heterogeneity, both the subgroup analyses and meta-regression were performed. They showed that the Year of publication (earlier than 2020 vs later than 2020), Race (European vs. non-European) and FMT intervention methods (oral vs. non-oral) were not associated with the heterogeneity. However, the results of the sensitivity analysis showed the study of Vrieze was the source of heterogeneity. The reason for this analysis may be due to the fact that among the included patients, the mean weight of patients in the allogenic group was significantly higher than that of patients in the autologous group (123kg vs 113kg), and there was no significant change in mean weight in both groups at the end of the 6-week trial (122kg vs 113kg). In the other included studies, there was no significant difference in the mean weight of patients in the two groups. This study was not excluded because of its greater weight and higher quality.

This is a systematic review and meta-analysis to assess the role of FMT in the treatment of obesity and metabolic syndrome. Compared to a previous meta-analysis, we included more studies to comprehensively analyze obesity parameters (e.g., weight, BMI), and metabolic system parameters (e.g., glucose, cholesterol). And we conducted a subgroup analysis of the two ways of using FMT to determine a more effective way of using it. There are several limitations to this systematic review. An important limitation is the small number of studies and patients, which leads to inconsistent and imprecise results, as well as large confidence intervals. In addition, this meta-analysis did not pre-register protocols (as in PROSPERO), which could introduce potential bias. A limitation of some studies is that diet and physical activity have not been tightly controlled. weight loss was associated with FMT treatment, and the beneficial effects of FMT may be negated by poor diet. It requires further study whether the addition of a standard dietary intervention could work synergistically with FMT donors to match host immunology better for optimizing clinical metabolic and immunological responses. A further dose finding study is needed to determine the optimal dose for this particular group of patients.

## Conclusion

In conclusion, FMT does not produce any serious adverse effects and may be beneficial as an adjunctive therapy in the treatment of metabolic syndrome, especially in improving blood glucose, increasing insulin sensitivity, and HDL cholesterol. However, due to the small number of relevant studies and the low quality of evidence, we need more high-quality studies on the role of FMT in the metabolic processes of glucose and lipids. Moreover, the fact that the FMT application is associated with changes in obesity-related parameters needs further confirmation when we include diet and lifestyle changes in the design.

## Supporting information

S1 Fig Funnel plot test for publication bias. (RAR)

S1 Table PRISMA flow diagram. (DOC)

S2 Table Summary of data for each study objective. (XLSX)

S3 Table Quality of evidence by Grading of Recommendations Assessment, Development and Evaluation (GRADE). (XLSX)

## List of abbreviations

BMI: body mass index
CDI: Clostridioides difficile infection
FBG: fasting blood glucose
FMT: fecal microbiota transplantation
HDL: high density lipoprotein
IBD: inflammatory bowel disease
IBS: irritable bowel syndrome
IR: insulin resistance
LDL: low density lipoprotein
MD: mean differences
MetS: metabolic syndrome
NAFLD: non-alcoholic fatty liver disease
RCT: randomized clinical trial
SCFA: short-chain fatty acid
SD: standard deviation
SMD: standardized mean differences
T2DM: type 2 diabetes mellitus
WMD: weight mean differences
